# Supplementary material for: Strongly Truncated Dnaaf4 Plays a Conserved Role in Drosophila Ciliary Dynein Assembly as Part of an R2TP-Like Co-Chaperone Complex With Dnaaf6
Source: Front Genet. 2022 Jul 6;13:943197. doi: 10.3389/fgene.2022.943197 (PMC9298768; doi:10.3389/fgene.2022.943197)
Supplement: Supplementary file 1 [file DataSheet1.PDF]

```
Fly      2 VQIS-----QTEEDIKISIELNRLVTRKPDVVLLPQYLKFNNPPIFFERHLAQEIDEMASFCRIF 61
           :|:|     ||:..:.:|:.|..:..|..||.....|||.|.||..||..|...||:.:|..:|.
Human    3 LQVSDYSWQQTKTAVFLSLPLKGVCVRDTDVFCTENYLKVNFPPFLFEAFLYAPIDDESSKAKIG 67

Fly     62 KNEARIVLVKKEKGLW---------PEMFQKLDKEALMQ---------------KR------LEI 96
           .:.....|.|||..:|         .||.|::.:::::|               ||      |.:
Human   68 NDTIVFTLYKKEAAMWETLSVTGVDKEMMQRIREKSILQAQERAKEATEAKAAAKREDQKYALSV 132

Fly     97 ADLIVERNKKRDE-----------KALERY-DNKRRAEIQKEIQRETDMRERVKQFQENSVREAL 149
           ...|.|..:|:.|           ||||.: :.:|:||.||:||||..:.::.||.:|.
Human  133 MMKIEEEERKKIEDMKENERIKATKALEAWKEYQRKAEEQKKIQREEKLCQKEKQIKEE------ 191

Fly    150 VVDVRKEAK---ATPKPDTLQYPPSSGGASRLAT-PLMRPPMSSVRGSGRINVNFTTQHKRVTPK 210
               ||:.|   .|....:....|....:..:.| .|....:.:.|..|.|.:|||   .||.|.
Human  192 ----RKKIKYKSLTRNLASRNLAPKGRNSENIFTEKLKEDSIPAPRSVGSIKINFT---PRVFPT 249

Fly    211 --RESQAAMEKAY 221
             ||||.|.|:.:
Human  250 ALRESQVAEEEEW 262
```

Supplementary Figure S1. Alignment of *Drosophila* and human DNAAF4 protein sequences. Alignments were produced by DIOPT analysis. The CS domain is shown in blue. Note that evidence of conservation extends beyond the CS domain. The alignment ends at human protein residue 262, such that the C-terminal region 263-420 (which contains the TPR domain) is not present in the *Drosophila* orthologue.
